# Mindful self-care and mental health among workers

**DOI:** 10.1192/j.eurpsy.2025.2012

**Published:** 2025-08-26

**Authors:** A. Abbes, I. Sellami, A. Feki, S. Baklouti, M. L. Masmoudi, M. Hajjaji, K. Jmal Hammami

**Affiliations:** 1 Occupationnal medecine; 2 Rheumatology, CHU Hedi Chaker, Sfax, Tunisia

## Abstract

**Introduction:**

Self-care refers to actions individuals take to maintain their health and prevent or manage illness. It increases awareness and offers a chance to improve workers’ quality of life. Mental health plays a key role in influencing self-care behaviours among workers.

**Objectives:**

Our study aims to assess self-care behaviours and mental health among workers.

**Methods:**

We conducted a descriptive, analytical, cross-sectional survey among workers. We collected sociodemographic, professional characteristics and self-care behaviours using Mindful Self-Care Scale (MSCS). Anxiety and depression were assessed using the Hospital anxiety and depression scale (HADS).

**Results:**

Our population comprised 116 female workers with a mean age of 41.5 ± 10.9 years. The mean of tenure of job was 12.8 ± 9.5 years. According to the HADS, the mean score of anxiety and depression were respectively 7.7 ± 5.2 and 7.9 ± 4.With the reference to HADS, 20 (17.2%) and 27 (23.3%) participants presented symptoms of anxiety and depression, respectively. Regarding the patients’ anxiety levels, it was found that 50%, 32.8%, and 17.2% appeared to have mild, moderate, and severe anxiety, respectively. As for the depression levels of patients, 56 were mildly depressed (48.3%), 33 were moderately depressed (28.4%), and 27 were severely depressed (23.3%). The mean of mindful self-care score was 99.2 ± 34.6. The mean of mindful relaxation, physical care, self-compassion and purpose, supportive relationships, supportive structure and mindful awareness were respectively 12.4 ± 5.2, 19.3±4.9, 20.7±8.9, 16.1±7.3, 13.2±6.3 and 13.8±4.7. Mindful relaxation and physical care were negatively correlated with anxiety (p = 0.01, r = -0.2).

**Conclusions:**

Our study found that workers with lower anxiety levels were more likely to engage in self-care. In the workplace, this leads to increased productivity and reduced attrition, as healthy employees are more productive and satisfied with their jobs. Therefore, providing mental health training for workers is essential to ensure their well-being.

**Disclosure of Interest:**

None Declared

